# CD4 lymphocyte dynamics in Tanzanian pulmonary tuberculosis patients with and without hiv co-infection

**DOI:** 10.1186/1471-2334-12-66

**Published:** 2012-03-21

**Authors:** Aase B Andersen, Nyagosya S Range, John Changalucha, George PrayGod, Jeremiah Kidola, Daniel Faurholt-Jepsen, Henrik Krarup, Harleen MS Grewal, Henrik Friis

**Affiliations:** 1 Department of Infectious Diseases, Odense University Hospital, University of Southern Denmark, Sdr. Boulevard 29, DK 5000 Odense C, Denmark; 2 National Institute for Medical Research, Muhimbili Medical Research Centre, Dar es Salaam, Tanzania; 3 National Institute for Medical Research, Mwanza Medical Research Centre, Mwanza, Tanzania; 4 Department of Human Nutrition, Faculty of Life Sciences, University of Copenhagen, 1958 Frederiksberg C, Denmark; 5 Department of Clinical Biochemistry, Aalborg University Hospital, 9000 Aalborg, Denmark; 6 The Gade Institute, Section for Microbiology and Immunology, University of Bergen and Haukeland University hospital, N- 5016 Bergen, Norway

**Keywords:** Pulmonary tuberculosis, HIV, CD4 cells, TB treatment

## Abstract

**Background:**

The interaction of HIV and tuberculosis (TB) on CD4 levels over time is complex and has been divergently reported.

**Methods:**

CD4 counts were assessed from time of diagnosis till the end of TB treatment in a cohort of pulmonary TB patients with and without HIV co-infection and compared with cross-sectional data on age- and sex-matched non-TB controls from the same area.

**Results:**

Of 1,605 study participants, 1,250 were PTB patients and 355 were non-TB controls. At baseline, HIV was associated with 246 (95% CI: 203; 279) cells per μL lower CD4 counts. All PTB patients had 100 cells per μL lower CD4 counts than the healthy controls. The CD4 levels were largely unchanged during a five-month of TB treatment. HIV infected patients not receiving ART at any time and those already on ART at baseline had no increase in CD4 counts after 5 months of TB treatment, whereas those prescribed ART between baseline and 2 months, and between 2 and 5 months increased by 69 (22;117) and 110 (52; 168) CD4 cells per μL after 5 months.

**Conclusions:**

The increase in circulating CD4 levels observed in PTB in patients is acquired after 2 months of treatment irrespective of HIV status. Initiation of ART is the strongest factor correlated with CD4 increase during TB treatment.

**Trial registration number:**

Clinical trials.gov: NCT00311298

## Background

The total number of circulating CD4 cells in HIV infected patients have in large patient series been acknowledged as the strongest, single predictive factor of clinical deterioration [1-3]. In individuals with latent *Mycobacterium tuberculosis *infection, CD4 depletion accelerates the progression from latent infection to active tuberculosis (TB), which, in turn, is believed to further fuel HIV replication rates due to elevated levels of pro-inflammatory cytokines [4]. TB by itself has also been associated with transitory lymphopenia including the CD4 positive cell lines [5,6]. A recent, retrospective study from Italy showed an impaired immune recovery in TB/AIDS cases compared to AIDS caused by other co morbidities which seemed not the be retrieved even after 3 years and despite access to efficient antiretroviral therapy (ART) [7]. However, the number of studies is small and some of the results are conflicting. In this prospective cohort of newly diagnosed pulmonary TB patients from an HIV endemic, sub-Saharan setting we report a large dataset studying the CD4 lymphocyte dynamics during TB treatment; both in HIV uninfected and HIV infected TB patients. Further, as this study was initiated before the general recommendation of initiating ART in TB-HIV co-infected patients already during active TB treatment, the majority of the HIV infected TB patients were not receiving ART.

## Methods

### Study design

The study was conducted from April 2006 to March 2009 in Mwanza, Tanzania. Pulmonary TB (PTB) patients were enrolled at four TB clinics in Mwanza city as part of a clinical trial on nutrition in PTB patients (clinical trial registration number NCT00311298 accessible at http://clinicaltrials.gov/ct2/show/NCT00311298, after giving informed consent [8,9]. The primary outcome of the study was weight gain at 2 and 5 month of intervention with energy and micronutrient enriched biscuits administrated through the initial 2 months of the TB treatment. A secondary outcome measure was to study the CD4 levels at 2 and 5 months. Pregnancy, age under 15 years, or terminal illness led to exclusion. The diagnosis of TB followed the World Health Organization (WHO) guidelines [10]. All TB suspects were asked to bring three sputum samples for smear microscopy after Ziehl-Nielsen stain, a fourth sample was obtained for culture on Loewenstein-Jensen substrate and the patients were asked to have a chest X-ray as appropriate [10,11]. Patients were classified as PTB+ if *Mycobacterium tuberculosis *could be cultured from the sample. In case of a negative culture or contamination, the diagnosis relied on positive microscopy of two samples or one microscopy positive sample and a chest X-ray suggestive of TB. A patient was considered PTB-smear-negative (PTB-) if all samples were culture and microscopy negative, but the TB diagnosis retained because chest X-ray was suggestive of TB, and there was no clinical response to a course of broad-spectrum antibiotics. After diagnosis, all patients were prescribed a standardized TB treatment for 6-8 months based on existing national guidelines [10,11]. The management of HIV infection was based on national guidelines at the time of the study [12]. Patients were supposed to start antiretroviral therapy (ART) if they had CD4 count of < 200 cells per μL, those with WHO stage 4 illness and/or CD4 count of 200-350 cells per μL were supposed to start ART after completion of 2 months of TB treatment. Patients who developed TB after starting ART continued ART throughout TB treatment.

### Recruitment of non-TB controls

The city of Mwanza is divided into wards, streets and communal cells. Each cell holds 10-20 households, and is headed by a so-called "ten cell-leader". Each of the PTB+ patients enrolled was asked to provide his/her residential address and the name of his/her ten-cell leader. The study team requested the ten-cell leader to provide a complete list of individuals in his/her jurisdiction meeting the age- and-sex recruitment criteria. Of these, one was randomly selected using a lottery method and invited to participate in the study as a non-TB control if he/she met the following criteria: no history of previous active TB or TB treatment, no evidence of current active TB (absence of cough, intermittent fevers, and excessive night sweating in the past 2 weeks and absence of unexplained weight loss in the past month), same sex as index case, aged 15 years or above and age difference from index case was not more than five years, had lived in the same street as index case for at least 3 months, not pregnant, and consenting to participate in the study. Persons who were terminally ill were not invited.

### Data collection

Data on demography, smoking, and alcohol intake were collected using questionnaires while data on ART were retrieved from antiretroviral-use databases in ART clinics. Between 8 and 12 a.m., blood was collected for HIV testing and for CD4 cell quantifying. HIV status was determined using "Capillus HIV-1/HIV-2" (Trinity Biotech Plc., Wicklow, Ireland) and "Determine HIV-1/HIV-2" (Inverness Medical Innovations, Inc., Delaware, USA) tests in parallel. HIV infection was diagnosed if both tests gave a positive result and an HIV negative diagnosis was made if both tests gave a negative result. Indeterminate results were resolved using ELISA "Organon Uniform II" (Organon Teknia Ltd, Boxtel, the Netherlands). CD4 counts were determined as cells per μl using a "Partec Cyflow Counter" (Partec GmbH, Münster, FRG) using the reagents suggested by the manufacturer: "Partec CD4 easy count kit 05-8401".

### Statistical methods

Data were double entered in EpiData (EpiData Association, Denmark) and analysed using Stata/IC version 11.2 (StataCorp, TX, USA). Normal probability plots were used to assess normality of continuous variables. Differences in categorical and continuous variables between groups were tested using chi-square test and t test or oneway analyses of varience, respectively. If the oneway analyses of variance was significant, then Scheffe multiple-comparison post hoc tests for differences between groups was done. Linear regression wasused to adjust for potential confounders and to test for interactions. P-values < 0.05 were considered significant.

### Ethical considerations

Ethical permission was obtained from the National Medical Research Coordinating Committee of the National Institute of Medical Research in Tanzania and approval from the Danish National Committee on Biomedical Research Ethics. Written and oral information was presented to all eligible participants before written consent was obtained. Written consent was obtained from parents/legal guardians of any participants under 18 years of age. Patients were offered pre- and post-test HIV counseling and referred to nearby antiretroviral clinics for management if they tested positive.

## Results

A total of 3,397 patients were eligible for the study, but 201 were below 15 years of age, 484 had extra-pulmonary TB, 49 were pregnant, 113 considered terminally ill and 1,239 were non-residents of the area. 61 patients were eligible but refused consent leaving 1,250 PTB patients to be included in the study. A total of 355 healthy controls were recruited from the neighborhood area and included as non-TB controls. Background characteristics are shown in Table 1. There were no differences in mean age between PTB+ patients and non-TB controls (0.8 years, 95% CI: -0.7; 2.3), since controls were selected among neighbors with same sex and similar age to PTB+ index cases. However, the mean age was 2.5 (95% CI: 1.04; 4.03) years higher in all PTB patients compared to non-TB controls. This was due to a 5.0 (95% CI: 3.5; 6.5) years higher mean age in PTB- compared to PTB+ patients. The prevalence of HIV infection was 50.6% (633) among the 1,250 PTB patients, and 9.9% (35) among the 355 non-TB controls (*p *< 0.001).

**Table 1:** Background characteristics of 1,250 pulmonary tuberculosis (TB) patients and 355 neighborhood controls ^1^

	Pulmonary TB patients (n = 1250)	Controls (n = 355)	P
Age (y)	36.5 (35.7; 37.2)	33.9 (32.7; 35.2)	< 0.001

Female sex, % (n)	40.8 (510)	45.4 (161)	0.13

Ethnicity, % (n)			

Wasukuma	45.6 (570)	46.3 (164)	0.82

Marital status, % (n)			

Single	24.8 (308)	25.2 (89)	< 0.001

Married/cohabiting	53.1 (658)	68.8 (243)	

Separated/divorced/widowed	22.1 (274)	6.0 (21)	

Occupation, % (n)			

Farmer/fisherman	39.1 (488)	32.2 (114)	0.06

Business/employed	36.1 (450)	40.4 (143)	

Other	24.8 (309)	27.4 (97)	

HIV infection, % (n)	50.6 (633)	9.9 (35)	< 0.001

^1 ^Pulmonary TB status was based on culture, except where culture data were not available. For each of 355 consecutive sputum positive TB patients an age- and sex-matched neighborhood control was selected. Data or mean (95% confidence interval) or % (n).

### CD4 levels before TB treatment

Data on CD4 counts were available on 1,604 (99.9%) of the 1,605 participants. The mean CD4 count was 416 (95% CI: 399; 433) cells per μL in PTB patients and 631 (95% CI: 595; 667) cells per μL in non-TB controls (Table 2). Thus, the CD4 count was 215 (95% CI: 178; 253) cells per μL lower among PTB patients compared to controls, partly due to confounding by HIV. Among HIV uninfected participants, the mean CD4 count was 105 (95% CI: 59; 151) cells per μL lower in PTB patients, and in HIV infected participants it was 128 (95% CI: 57; 198) cells per μL lower (Table 2). Accordingly, in linear regression analyses, there was no interaction between PTB and HIV status with respect to CD4 counts (interaction, *p *= 0.68).

**Table 2:** CD4 counts among 1,250 pulmonary tuberculosis (TB) patients and 355 neighborhood controls^1^

	TB patients (n = 1250)	Controls (n = 355)	Difference (controls - TB)	P
CD4 counts (cells per μL)	416 (399; 433)	631 (595; 667)	215 (178; 253)	< 0.001

In HIV uninfected	550 (523; 577)	655 (617; 693)	105 (59; 151)	< 0.001

In HIV infected	285 (269; 301)	413 (330; 495)	128 (57; 198)	< 0.001

^1 ^Pulmonary TB status was based on culture, except where culture data were not available, in which case microscopy results were used. From each of 355 consecutive sputum positive TB patients an age and sex-matched neighborhood control were selected.

The independent effects of PTB and HIV status were estimated in the multivariate model, while adjusting for age and sex (Table 3). Age was adjusted for using dummy variables with below 25 years as reference category. Ages between 25 and 35, 35-45 and 45 years and above were associated with 114, 106 and 71 cell per μL lower CD4 count compared to the age below 25 years. Sex was not a predictor of CD4 count (95% CI: -45; 15). As seen, both PTB+ and PTB- status was associated with around 100 cells per μL lower CD4 counts, while HIV infection was associated with around 250 cells per μL lower CD4 count. The intercept of 736 (95% CI: 695; 778) reflects the mean CD4 count among individuals falling into all the reference categories; i.e. female non-TB controls without HIV infection and age below 25 years.

**Table 3:** Predictors of CD4 count (cells per μL) among 1,250 pulmonary tuberculosis (TB) patients starting TB treatment and 355 controls with regression coefficients B, 95% confidence interval (95% CI) and p-values^1^

	Univariate		**Multivariate **^**2**^	
	**B (95% CI)**	**P**	**B (95% CI)**	**P**

Pulmonary TB status				

Controls (n = 355)	-		-	

PTB negative (n = 427)	-247 (-292; -203)	< 0.001	-103 (-147; -58)	< 0.001

PTB positive (n = 822)	-199 (-238; -160)	< 0.001	-109 (-146; -71)	< 0.001

HIV status				

Uninfected (n = 937)	-		-	

Infected (n = 667)	-294 (-323; -265)	< 0.001	-246 (-279; -215)	< 0.001

^1 ^Pulmonary TB status was based on culture, except if culture data were missing in which case microscopy results were used. From each of 355 consecutive PTB positive TB patients, an age- and sex-matched neighborhood control was selected, and used as reference category.

^2 ^Adjusted for sex and age. Age was included as dummy variable with below 25 years as reference category. N = 1604, R^2 ^= 0.23, intercept 736 (95% CI: 695; 778).

### CD4 levels during TB treatment

**Figure 1:**
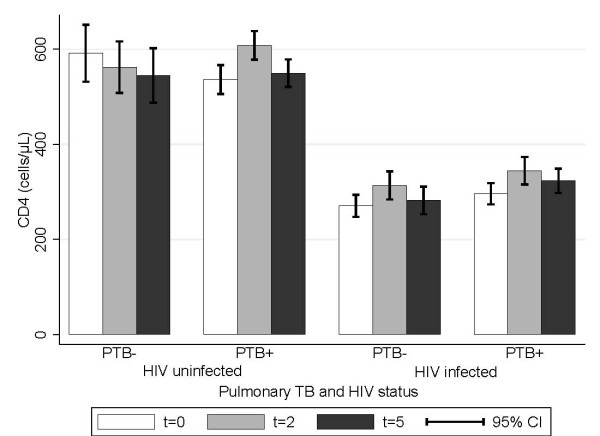
**CD4 levels at different time points during TB treatment**. PTB +/-; pulmonary TB case microscopy or culture positive/negative. Time is indicated in months: e.g. t = 2; sample drawn after 2 months of terapy.

Of the 1,250 PTB patients, 1,119 (89.6%) had their CD4 cells determined again after 2 months and 1,020 (81.6%) after 5 months of treatment. The changes in CD4 count during TB treatment seemed to depend on PTB and HIV status (Figure 1). As seen in Table 4, among HIV uninfected PTB- patients the changes in CD4 count were -39 (95% CI: -106; 28) after two and -60 (95% CI: -143; 22) cells per μL after 5 months of treatment. In contrast, HIV infected PTB+ patients had a significant increase after 2 months (78 cells per μL, 95% CI: 39; 117), which disappeared after 5 months (14 cells per μL, 95% CI: -28; 55). At 2 months, the change in CD4 count in HIV uninfected was 118 (95% CI: 37; 199) cells per μL higher in PTB+ compared to PTB- patients (*p *= 0.004). Among HIV infected PTB patients, the changes in CD4 count were similar to that of HIV uninfected PTB+ patients, i.e. significant, but transient increments in CD4 counts (Table 4**)**. However, in HIV patients ART status and the timing of initiation of ART were major determinants of the change in CD4 count during TB treatment.

**Table 4:** CD4 counts (cells per μL) in 1250 pulmonary tuberculosis (TB) patients by HIV and sputum status^1^

	HIV uninfected (n = 617)		HIV infected (n = 633)		
	**PTB- (n = 151)**	**PTB+ (n = 466)**	**PTB- (n = 276)**	**PTB+ (n = 357)**	**P**

Baseline^2 ^(n = 1249)	592 (532; 651)^a, b^	536 (506; 566)^c, d^	271 (248; 294)^a, c^	296 (274; 318)^b, d^	< 0.001

2 months (n = 1119)	562 (508; 616)^a, b^	608 (578; 638)^c, d^	313 (284; 342)^a, c^	344 (316; 373)^b, d^	< 0.001

Change	-39 (-106; 28)^a^	78 (39; 117)^a^	34 (3; 64)	50 (19; 80)	0.009

5 months (n = 1020)	545 (488; 602)^a, b^	549 (521; 578)^c, d^	282 (254; 311)^a, c^	323 (298; 348)^b, d^	< 0.001

Change	-60 (-143; 22)	14 (-28; 55)	2 (-31; 34)	26 (-5; 57)	0.16

^1 ^Data are mean (95% confidence interval). Where p-values are found significant, means with different suprascripts are significantly different. Pulmonary TB status was based on culture, except where culture data were not available, in which cases microscopy results were used. ^2 ^CD4 results missing for one patient.

According to questionnaire data as well as data from the ART clinic registers, 80 (12.6%) of the 633 PTB patients with HIV co-infection were on ART at the time they started TB treatment. The remaining 553 (87.4%) were all referred to ART clinics. Two and five months after start of TB treatment 167 (26.4%) and 222 (35.1%), respectively, were on ART. However, not all came for their two and five-month follow-up visits. Of the 546 (86.3%) HIV infected who came for their 2 months follow-up visit and had their CD4 count determined, 144 (26.4%) were on ART. Similarly, among 500 (79.0%) HIV infected examined at 5 months, 183 (36.6%) were on ART.

The changes in CD4 count during TB treatment among the 633 HIV co-infected PTB patients are shown in Table 5 by the four categories defined by ART status and timing of ART initiation. The four categories are: 1) not on ART at any time during the study, 2) on ART at baseline, 3) put on ART between the baseline and the 2 month examination and 4) on ART between the 2 and 5 months examination. As seen, the CD4 counts differed significantly between these four categories at baseline and after 2 months, but not after 5 months. At baseline, those who were not put on ART (prior to the 5 months follow-up examination) had the highest mean CD4 count. Of the remaining three categories, the CD4 count was highest in those already on ART, and lowest in those put on ART between the baseline and 2 months examination.

**Table 5:** Change in CD4 counts (cells per μL) during tuberculosis (TB) treatment in 633 HIV positive pulmonary TB patients by timing of antiretroviral treatment (ART) in relation to start of TB treatment ^1^

	Not on ART (n = 410)	ART at baseline (n = 80)	ART 0-2 months (n = 87)	ART 2-5 months (n = 55)	P
Baseline^2 ^(n = 632)	315 (293; 336)[410]^a^	262 (223; 300)[80]	196 (167; 225)[87]^a^	238(197;279)[55]^a^	< 0.001

2 months (n = 546)	361 (333; 389)[348]^a^	299 (253; 345)[72]	261 (216; 306)[72]^a^	283 (231;335)[55]	0.002

Change	40 (10; 70)[348]	41 (-13; 96)[72]	60 (14; 106)[72]	44 (-10; 99)[55]	0.95

5 months (n = 500)	314 (289; 339)[317]	286 (248; 323)[64]	268 (217; 319)[67]	344 (282;405)[52]	0.20

Change	-11 (-42; 20)[317]^a^	20 (-31; 70)[64]	69 (22; 117)[67]	110 (52; 168)[52]^a^	0.004

^1 ^Data are mean (95% confidence interval). Where p-values are found significant, means with different suprascripts are significantly different. Pulmonary TB status was based on culture, except where culture data were not available, in which cases microscopy results were used.

The changes in CD4 count up to 2 months did not differ between the four groups (Table 5). In contrast, the changes from baseline to 5 months were different between the groups. In fact, only those prescribed ART between baseline and 2 months (69 cells per μL; 95% CI: 22; 117) and between 2 and 5 months (110 cells per μL, 95% CI: 52; 168) had sustained increments in CD4 counts.

## Discussion and Conclusions

This study shows that the decrease in circulating CD4 lymphocytes induced by TB has occurred before the diagnosis is made. The pattern was the same for both TB patients with and without HIV co-infection. HIV uninfected PTB patients had significantly lower CD4 levels than healthy controls at baseline and did not reach the same levels of circulating CD4 cells even after 5 months of TB treatment. This could either be explained by continued sequestering of cells to the lungs or due to apoptosis and persistent regulatory stimuli even at this late stage towards the end of treatment [13-15]. The HIV infected TB patients who were already on ART at time of TB diagnosis likewise did not increase their pool of circulating CD4 cells during the 5 months observation and treatment period. However, the HIV patients either put on ART within the first 2 months or from the second to the fifth month, experienced an increase in CD4 lymphocytes of 69 (95% CI: 22; 117) and 110 (95% CI: 52; 168). A weakness of our study is the lack of data regarding symptoms of Immune Reconstitution Inflammatory Syndrome (IRIS), which might have explained some of the CD4 fluctuations. The incidences of IRIS have been variably reported but higher in patients receiving early ART and in patients with low CD4 counts [16,17].

The data were analysed separately for PTB+ and PTB- patients because the PTB- patients probably represent a rather inhomogeneous group. These patients may range from HIV uninfected individuals with early clinical manifestations of pulmonary TB to severely immune suppressed HIV infected patients who are excreting too few bacteria to be detected in ordinary sputum samples. There was no access to enhanced diagnostic procedures like induced sputum maneuvers or bronchoscopy in this setting. A subgroup of the patients may not even have TB but have pulmonary symptoms for other reasons even though the criteria for initiating TB treatment in smear negative patients according to WHO guidelines were followed. It was a matter of concern whether some of the smear negative HIV patients were in fact suffering from *Pneumocystis jirovecii *pneumonia, but a nested study performed in the same cohort including approximately one third of the study participants from the present study did not confirm this suspicion [18]. However, the persistent decline in CD4 cells in the HIV uninfected, PTB- group could suggest that these patients have other co-morbidities. The prognosis and diagnostic set up for this category of patients should be further studied.

Some of the early descriptions of TB patients with CD4 lymphocyte depletion were case series describing few, severely ill and hospitalized TB patients [6]. A study from South Africa from 1995 reported the CD4 profiles of 241 HIV uninfected and 154 HIV infected hospitalized TB patients during the initial 3 months of TB treatment [19]. In that study the CD4 count increased in both the HIV infected and the HIV uninfected group receiving TB treatment; although at slowest rates and to lower levels in the HIV infected groups. It was noted that the defaulter rate was more than 50%, which may have introduced a bias. A study from 1997 conducted in the USA included 85 HIV uninfected TB patients and half of these patients had subnormal CD4 levels [5]. In this study low CD4 levels were associated with disease severity markers like low serum albumin, body weight, low haematocrit and extensive pulmonary disease. The analyses were repeated after 1 month of TB treatment and most patients had obtained normal CD4 levels at that time. A study from Senegal including TB patients from 1995 to 1996 found that among 430 HIV negative patients 14.4% had a CD4 count below 300 cells per μL [20]. There are no follow up data presented from this cohort.

In a more recent study from South Africa assessing 111 HIV infected pulmonary and extra-pulmonary TB patients recruited from 1997 to 1998 [21] also suffered from a quite high defaulter rate leaving only data from 57 patients (51%) for the final analyses. The main conclusion of this paper was that viral loads are high and remains high throughout the TB treatment period. However, CD4 cell numbers were relatively stable, slightly increasing tendency during the observation period, which was interpreted as a positive effect of the TB treatment. This finding is in line with results obtained from patients from Uganda [22] in which 38 HIV infected sputum smear positive TB patients with an initial CD4 count > 350 cells per μL were followed for 12 months. TB therapy clearly ameliorated the signs of immune activation, but HIV viral loads and CD4 levels remained unchanged throughout the study period. A recently published, retrospective study from Italy including 125 HIV coinfected TB patients (both pulmonary and extra pulmonary TB) found an impaired immune recovery of these patients compared to non-TB HIV patients which for some of the patients was persistent even after 3 years [7]. The authors found an association to delay in viral suppression in the HIV-TB patients group.

The strength of our study was the inclusion of randomly selected non-TB controls at baseline, and the large number of TB patients and high follow-up rates, i.e. 90 and 80% at 2 and 5 months, respectively. Therefore, we were able to compare the immune status of the 1,250 PTB patients to that of 355 non-TB controls. The HIV prevalence of the control group was around 10% and the CD4 levels in this group was as expected higher than in the HIV infected TB patient group. However, the mean CD4 count of 285 cells per μL (95% CI 269;301) of the HIV infected TB patients indicate that these were patients with only moderately progressed HIV infection reflecting that the study population was enrolled from outpatient clinic settings, not including severely ill patients requiring admission.

The latest recommendations from the WHO advocate that ART should be initiated in HIV patients with active TB "as soon as possible within 8 weeks after the start of TB treatment"[3]. The authors of the guidelines state this as a "strong recommendation". Data from prospective clinical studies addressing early versus deferred ART of HIV positive TB patients clearly demonstrates an improved survival especially in HIV-TB patients with very low CD4 levels [16,17]. Our data confirm the beneficial effects of early ART on CD4 recovery but also show that the immune restoration even in HIV uninfected patients has not fully taken place even when the TB treatment is almost completed after 5 months treatment. The biological implications of this warrant further studies.

## Competing interests

The authors declare that they have no competing interests.

## Authors' contributions

ABA: Design of study, interpretation of results, manuscript preparation and submission. RNS: Design of study, interpretation of results, drafting of manuscript. CJ: Design of study, local coordination of project, drafting of manuscript. PGJ: Collection of samples, local coordination of project, drafting of manuscript. KJ: Collection of samples, local coordination of project, drafting of manuscript. F-JD: collection of samples, data analysis, drafting of manuscript. KH: Data analysis and drafting of manuscript. GH: Data analysis and drafting of manuscript. FH: Design of study, interpretation of results and manuscript preparation. All authors read and approved the final manuscript.

## Pre-publication history

The pre-publication history for this paper can be accessed here:

http://www.biomedcentral.com/1471-2334/12/66/prepub
